# HIV-1 envelope characteristics that coincide with the development of cross-reactive neutralizing activity in HIV-1 infected patients

**DOI:** 10.1186/1742-4690-8-S2-P69

**Published:** 2011-10-03

**Authors:** Tom van den Kerkhof, Marit van Gils, Zelda Euler, Naomi Verwer, Rogier Sanders, Hanneke Schuitemaker

**Affiliations:** 1 Dept of Exp Immunology and Landsteíner Laboratory of the Academic Medical Center at the University of Amsterdam, Amsterdam 1105 AZ, The Netherlands; 2 Dept of Med Microbiology and Experimental Virology of the Academic Medical Center at the University of Amsterdam, Amsterdam 1105 AZ, The Netherlands

## Background

The HIV-1 envelope glycoprotein (Env) on the viral surface is the target for anti-HIV-1 neutralizing antibodies. In some HIV-1 infected patients broadly neutralizing antibodies (BrNAb) develop that neutralise HIV-1 from different subtypes and therefore are considered to be directed against conserved regions [2]. The most vulnerable epitopes can be shielded from antibody recognition by steric hindrance and N-linked glycans [3]. Moreover, length and glycosylation of the envelope V1V2 region seem to play a role in resistance against neutralizing antibodies [4]. Understanding of factors involved in BrNAb development will help the rational design of an effective antibody-based vaccine immunogen.

## Methods

From a previous screening of 299 patients for broadly neutralizing activity (BrNAc), we selected 13 patients with high BrNAc in serum, 9 patients without BrNAc (non-BrNAc) and 10 patients with intermediate BrNAc, at 2-4 years after SC. From these patients clonal viruses were obtained within the first year after SC from which gp160 was sequenced and analyzed for length of different regions and number of potential N-linked glycosylation sites (PNGS). Additionally we tested the neutralization sensitivity of the viral variants from the patients with and without BrNAc against different already identified broadly neutralizing antibodies (BrNAbs).

## Results

We observed a correlation between length of the variable regions 1 and 2 (V1V2) of *env*, especially for variable region 1 (V1), and the presence of BrNAc. Patients with non-BrNAc in their serum had HIV-1 variants with longer V1 regions. Remarkably, differences in length of the V1 region did not coincide with differences in the number of PNGS on V1. Only the number of PNGS in gp41 correlated with BrNAc, where a higher number of PNGS correlated with the absence of BrNAc. No significant differences were observed between HIV-1 variants from individuals with and without BrNAc in their sensitivity to the known BrNAbs b12, 2G12, 2F5, 4E10, PG9, PG16 and VRCO1.

## Conclusion

The observed correlation between a shorter V1 of *env* in patients with BrNAc could indicate that the development of BrNAc in HIV-1 infected patients is associated with a more accessible and more open structure of the viral envelope glycoprotein, which is achieved by having fewer glycans and shorter variable loops. However these *env* characteristics on early viruses isolated from patients who developed BrNAc did not coincide with a higher sensitivity to BrNAbs as compared to early viruses from patients who did not developed BrNAc. Identification of *env* characteristics that coincide with BrNAc in patients could help the rational design of an effective antibody-based vaccine immunogen.
